# International Clinics

**Published:** 1901-05

**Authors:** 


International Clinics.—A quarterly of clinical lectures, and especially preferred articles on medcine, neurology, surgery, therapeutics, obstetrics, pediatrics, pathology, dermatology, diseases of the eye, ear, nose and throat, and other topics of interest to students and practitioners. By leading members of the medical profession throughout the world. Edited by Henry W. Cottell, A. M., M. D., Philadelphia, Director of the Ayer Chemical Laboratory of the Pennsylvania Hospital, with the collaboration of John Ashurst, Jr., M. D., LL. D., and Charles H. Reed, M. D., of Philadelphia; James T. Whittaker, M. D., LL. D., of Cincinnati, with regular correspondents in Montreal, London, Paris, Leipsic and Vienna. Volume II. Tenth series. 1900. 300 pages. Price, $3.00. Philadelphia: J. B. Lippincott Company. 1900.

The International Clinics has now reached its tenth consecutive year of publication, and is most popular with the profession. These quarterly books embody the polyclinic idea of teaching, the articles being in the form of clinical lectures, and are of the most practical value. The present volume contains the last literary work of the late John Ashurst, Jr., and James T. Whittaker.

The articles of this volume are thirty-five in number, and take a wide range. One is devoted to new inventions, seven to therapeutics, ten to general medicine, two to neurology, four to surgery, two to obstetrics, seven to diseases of the eye and ear, one to dermatology and one to the fifty-first annual meeting of the American Medical Association. T. J. B.

